# Study on the impact and attention of cover papers for PLOS journals: Evidence from propensity score matching

**DOI:** 10.1371/journal.pone.0329773

**Published:** 2025-08-27

**Authors:** Xiaoqing Chen, Li Liu, Weichao Li, Meiyuan Xing, Xiuyang Li

**Affiliations:** 1 Library, The First Affiliated Hospital, College of Medicine, Zhejiang University, Hangzhou, China; 2 Department of Big Data in Health Science, and Center for Clinical Big Data and Statistics, The Second Affiliated Hospital, College of Medicine, Zhejiang University, Hangzhou, China; Max Planck Society, GERMANY

## Abstract

**Background:**

Studies on the impact and attention of cover papers within open-access journals from the same publisher remain lacking. The objective of this study was to explore the effect of being featured as a cover paper on the impact and attention of papers for PLOS journals using propensity score matching (PSM).

**Methods:**

Cover and non-cover papers published in five PLOS journals (i,e., *PLOS Biology*, *PLOS Computational *Biology**, *PLOS Genetics*, *PLOS Pathogens*, *PLOS Neglected Tropical Diseases*) between 2008 and 2017 were selected. Three scientometric indicators, citations (as scholarly impact indicator), and Altmetric attention score (AAS) and Mendeley readers (as social and academic attention indicators) of each paper were collected from altmetric.com. Two datasets were obtained via 1:2 propensity score matching: one spanning 2008–2017 for analyzing citations and reader counts, and another covering 2011–2017 for AAS analysis. Then, the Wilcoxon signed-rank test, univariate analysis, and multivariate linear regression analysis were conducted to explore the impact and attention of cover papers versus non-cover papers.

**Results:**

Among 24,080 papers, 1,760 were successfully matched for analysis of citation frequency and readership, and 1,212 were successfully matched for Altmetric attention analysis. After PSM, cover papers exhibited significantly higher citations (*v* = 560, *P* < 0.001), much more readers (*v* = 528, *P* < 0.001), and higher AAS (*v* = 1384, *P* < 0.001) than non-cover papers. Further multivariate regression analysis of the PSM-adjusted data revealed significant associations, with regression coefficients of 0.145 for citations, 0.174 for readership, and 0.691 for AAS (*P* < 0.05).

**Conclusions:**

The findings of this study suggested that being featured on the cover was statistically positively associated with an article’s academic impact and public visibility, especially in terms of the societal attention. However, the relationship was weak.

## Introduction

The cover of a scientific journal is an important representation of the journal’s theme and the papers inside it. In the era of print, journal covers made the first impression on readers and were a significant aspect of a journal’s identity, providing an overview of journal articles and encouraging readers to pick up and delve into the publication [1]. With the rapid development of the digital age, consumers increasingly prefer to read online rather than print journals. Nevertheless, cover papers continue to be selected to be prominently featured on numerous journal websites that receive a lot of traffic (e.g., *Nature*, *Nature Chemistry*, *Emerging Infectious Diseases*, *Cell*, *Acta Naturae*).

There are various and complex reasons why a research image or paper is chosen to feature on the front cover of a journal: aesthetic quality of the image and scientific quality [2], technical reproducibility, stylistic continuity, communication effectiveness, audience appeal, importance, originality, interdisciplinary interest, timeliness, accessibility, elegance, surprising conclusions, or any other aspect [3,4]. Being featured on the cover of a prestigious journal is a highly sought-after achievement in the highly competitive world of scientific publishing. This can enhance recognition, status, and financing opportunities for a research endeavor. Several academic organizations and groups would even publish news about it on their official websites, citing the cover articles [5].

Currently, the question of whether cover papers receive higher scholarly impact and attention than non-cover papers has become a widely discussed topic. A cover paper is the research featured on the cover of a journal with images and simple words, while other papers are considered non-cover papers [6]. Wang et al. [7] compared the usage and citation data for PLOS Biology cover and non-cover articles from 2006 to 2010 and confirmed no significant difference in attention or impact. Wang et al. [8] compared the citation data between cover and non-cover papers in CNS journals (*Cell*, *Nature* and *Science*) from 2008 to 2010. Kong and Wang [6] compared citations and Altmetric scores between cover and non-cover papers in Nature. Wang et al. [1] analyzed the citations and utilizations of cover and non-cover papers published in 42 cell biology journals from 2011 to 2015. Wan et al. [9] compared the citations and download rates of cover and non-cover papers published in five Chinese pharmacy journals in 2019 and 2020. Based on the 30 years of bibliometric data, Battiston et al. [10] investigated how an article on the cover of the journal Nature affected citations to all articles written by its authors. Research on the effect of journal covers continues to evolve.

Previous studies have predominantly focused on prestigious journals with remarkably high impact factors, ranking in the top thirty among more than 10,000 journals in the Journal Citation Reports since 2010, and journals in the disciplines of cell biology and pharmacy, revealing that cover papers may draw more citations and attention than non-cover papers. However, a study focusing solely on PLOS Biology presented contrasting findings, revealing no significant difference in terms of attention or impact. In addition, existing studies have failed to distinguish the potential influence of open access (OA) from other publishing models (such as print, online, or dual publishing formats) on citations or other metrics, often conflating these different modes. To some extent, OA articles have increased the number of views, expanded the readership, and contributed to the dissemination of knowledge [11]. Research has reported that OA status is a significant, independent predictor of metrics like AAS and Mendeley readers [12]. Compared to the traditional subscription publishing route, OA publications may exhibit significantly higher AAS and citations [13,14]. Meanwhile, electronic journals were cited more frequently than paper journals in literature [15]. The duration of online-to-print lags has been shown to be associated with citations both before and after publication in journals that adopt a dual print-online publishing model [16]. One prior study discovered a statistically significant correlation between the utilization of online journals by subscribers and their citations in literature [17]. Considering these contexts, a new question arises: Can cover papers published in journals with the same publishing model, but possessing different (yet not remarkably high) impact factors, and spanning various disciplines generate a similar impact? This underscores the need for additional research in this area.

Presently, studies on the impact and attention of cover papers within open-access journals from the same publisher remain lacking. Thus, in this study, taking papers in five PLOS journals (i.e., *PLOS Biology*, *PLOS Computational *Biology**, *PLOS Genetics*, *PLOS Pathogens*, and *PLOS Neglected Tropical Diseases*) from the same publisher as an example, we explored the effect of being featured as a cover paper on the academic impact and attention for papers in journals without exceptionally high impact factors and spanning multiple disciplines, using the propensity score matching strategy.

## Methods

In this study, papers from five PLOS journals (i.e., *PLOS Biology*, *PLOS Computational *Biology**, *PLOS Genetics*, *PLOS Pathogens*, *PLOS Neglected Tropical Diseases*) were selected as the research object for following reasons: (1) They are open access with well-established peer-review criteria journals published by the same publisher, PLOS; (2) There are published in the same format; (3) They provide article-level metrics data for every article early which are adaptable to the altmetrics; (4) They are published monthly, and almost every issue features a cover paper of an original article or review because of its eye-catching or striking image; (5) They represent different disciplinary fields.

### Scientometric indicators

The frequency of citation is used to assess the quality or impact of an article. Web of Science (WOS) and Scopus are well-known citation indexes, in which most citation data is not freely accessible. Dimensions, a potential new source of impact data, is a partially free academic database for publications, grants, clinical trials, patents, and policy documents launched by Digital Science in January 2018 [18]. Currently, Dimensions is the largest and broadest index of scientific documents [19], including a higher coverage of publications, and similar citation counts to those in WOS and Scopus [20,21].

Altmetrics are metrics and qualitative data that complement traditional, citation-based metrics [22]. Altmetric.com was established in 2011 to comprehensively track and measure the Altmetric score indicators. The Altmetric attention score (AAS) is an indicator, reported by the so-called ‘Altmetric donut’, which uses an algorithm to calculate the amount of attention a specific paper has received in different social media (e.g., Facebook, Twitter, and Blogs) and online platforms (e.g., F1000 and peer reviews) [23]. The more attention such a scholarly article receives, the higher its AAS will be. Meanwhile, Altmetric.com includes data sources involving reference managers available online like Mendeley. Mendeley reader counts were valuable for the early impact evaluation of a study that reflected the influence of publications in terms of readership [24,25]. The AAS related analysis in this study was conducted on articles published since 2011.

According to the above description, three scientometric indicators, citations in Dimensions (as scholarly impact indicator), and AAS and Mendeley reader counts (as social and academic attention indicators) for both cover and non-cover papers were involved in this study. This study also included several control variables potentially correlated with these scientometrics, such as source journal, publication year, number of authors, reference count, countries, title length, international collaboration, number of pages, keywords, abstract length, affiliations, and funding sources [1,26]. As a key bibliographic indexing organization, WOS/Clarivate provides defined categories such as Biology, Geography, Geology, Philosophy, Physics and Psychology [27]. Each journal in this study represented a different research field within WOS. Therefore, the source journal, a key control variable, served as a proxy for the research field of the papers analyzed. Other controlled variables represented paper characteristics potentially related to the impact of articles [28,29].

### Data collection

**Fig 1:**
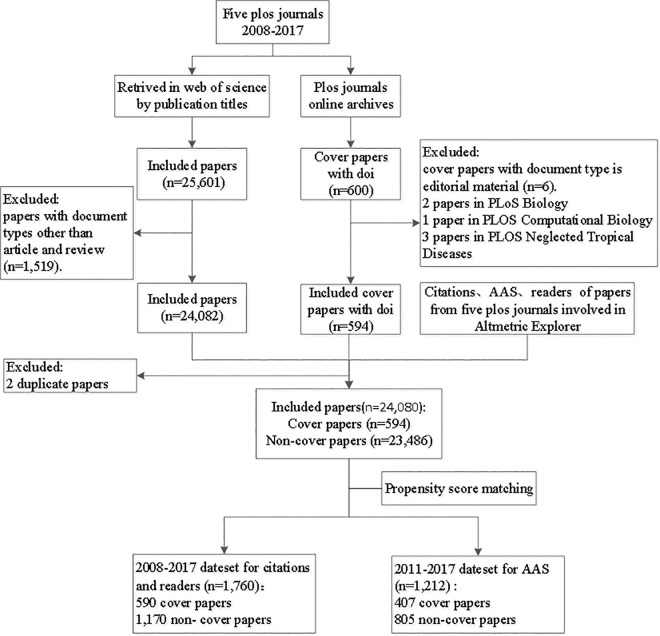
Illustration of the data collection procedure.

In this study, the Web of Science and the Altmetric Explorer were used as two major data sources. Fig 1 illustrated the data collection procedure. The titles and dois of each cover paper were collected from the PLOS journals website (https://plos.org/#journals). To allow sufficient time (2–5 years) for citations, papers published from 2008 to 2017 were selected. There were twelve PLOS journals in total, and 7 journals were excluded: *PLOS Medicine* was excluded because the article type of its cover paper was editorial material; *PLOS One* was excluded for its multidisciplinary nature and without cover paper; And the other 5 journals were not published until 2021.

Papers published between 2008 and 2017 in the 5 PLOS journals were exported from the WOS. And the document types were limited to “article” or “review”. Data on variables such as source journal, publication year, funding and collaborations, number of references, pages, keywords, abstracts, affiliations, authors, countries, and title length were extracted from the WOS or calculated based on the recorded information. The three scientometric indicators for this study were collected and downloaded from altmetric.com according to the journal title. For papers not included in altmetric.com, these indicators were assigned a value of zero. Cover papers were labeled as 1, and non-cover papers as 0. The three scientometric indicators and characteristics of each paper were matched using the DOI or title to integrate different datasets into one. All datasets were obtained on August 21, 2023.

### Data analysis

First, this study provided a brief description of all the obtained data.

Then, sample papers were selected for further comparative research using a propensity score matching (PSM) strategy. PSM, introduced by Rosenbaum and Rubin in 1984 [30], helps to mitigate the possible effects of selection bias or confounding bias that could occur during the analysis. The fundamental principle of PSM is to equilibrate the distribution of observed confounding covariates between the treated and untreated groups by matching individuals whose propensity scores are equal or very similar across the two groups [31,32]. The steps and calculation methods of PSM have been previously described in detail [33,34]. PSM has been recognized as a useful matching technique for scientometric studies [33,35]. The commonly used matching methods include nearest neighbor matching, caliper matching, and global optimal matching, etc. In this study, the number of papers in the cover and non-cover groups differs significantly, so the PSM method was used as a sampling technique. In PSM, we adopted the one-to-two nearest neighbor matching method and used calipers to control the maximum distance [36]. Propensity score analysis was performed using the “matchit” package in R as described by [37]. And chi-square or non-parametric tests were used to examine the performance of the matching. In this study, the outcome variable was the impact of papers. Citation counts were used to measure their academic impact, while AAS and readers were chosen as proxies for their societal visibility. The treatment variable was whether a paper was designated as a cover paper.

Finally, based on the samples obtained through PSM, a non-parametric Wilcoxon signed-rank test, univariate analysis, and multivariate linear regression analysis were conducted to compare the influence of the two groups. To reduce the skewness of the data, the natural logarithmic transformation ln(1 + χ) was applied to the data set [29,38,39]. The values of the variance inflation factor (VIF) across all models were consistently below 10, indicating the absence of multicollinearity among the variables in this study. Data were analyzed with R 4.3.1 and SPSS 26.0.0. *P* less than or equal to 0.05 was considered to be statistically significant.

## Results

Finally, 24,080 papers were collected after processing, including 594 cover papers and 23,486 non-cover papers in five PLOS journals (Table 1). Two cover papers in *PLOS Biology* [40,41], one in *PLOS Computational Biology* [42], and three cover papers in *PLOS Neglected Tropical Diseases* [43–45] were excluded for the article type of editorial material, so cover papers in these three journals were less than the other two.

**Table 1:** The number of cover and non-cover papers in 5 PLOS journals.

Journal	Total	Cover	Non-cover	5-year JIF^a^	JIF Quartile^a^
*PLOS Biology*	1,977	118	1,859	8.2	Q1
*PLOS Computational Biology*	4,769	119	4,650	3.8	Q1
*PLOS Genetics*	6,074	120	5,954	4.0	Q1
*PLOS Neglected Tropical Diseases*	5,477	117	5,360	3.4	Q1
*PLOS Pathogens*	5,783	120	5,663	5.5	Q1
All	24,080	594	23,486		

a: JIF = journal impact factor, the JIF was from Journal Citation Reports (Clarivate, 2023).

### Descriptive statistics

The non-normal distributions of citations, AAS, and the number of readers indicated that the median values of cover papers exhibited higher compared to those of non-cover papers (Table 2). The papers with the highest citations (5263) and AAS (12462), and most readers (5671) were published in *PLoS Biology* [46,47], which were all non-cover papers.

**Table 2:** Descriptive statistics for the citations, AAS, and Mendeley reader counts of all the cover and non-cover papers.

Variables	Period	Median(IQR)		
		All	Cover papers	Non-cover papers
Citations	2008 ~ 2017	45 (62)	60 (79.25)	45 (62)
Readers	2008 ~ 2017	77 (85)	102 (117.50)	77 (83)
AAS	2011 ~ 2017	4 (11)	12 (34.25)	4 (12)

### Propensity score matching

**Fig 2:**
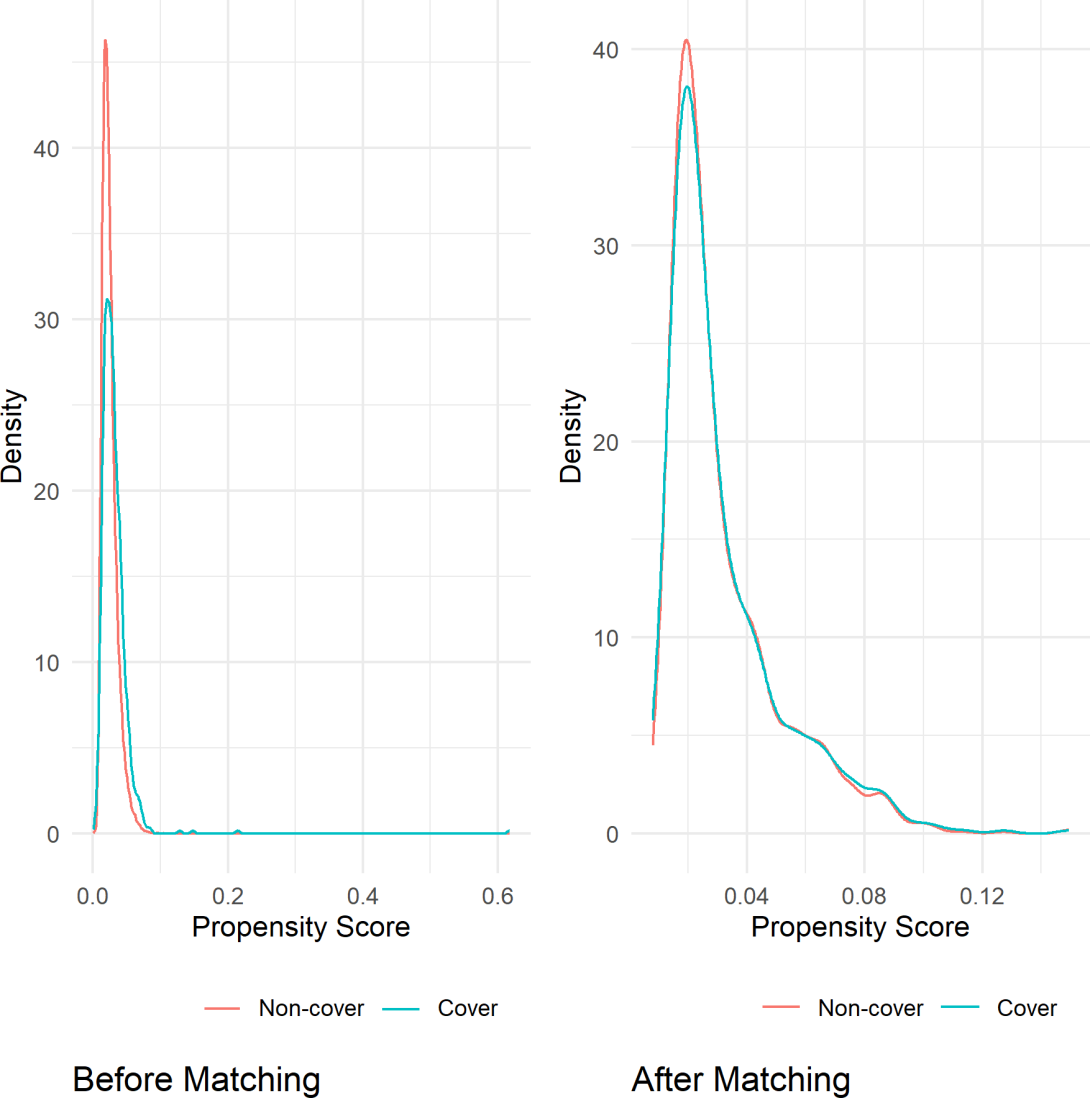
The kernel density of propensity score for the cover and non-cover groups before and after matching for the dataset analyzing citations and readers from 2008 to 2017.

**Fig 3:**
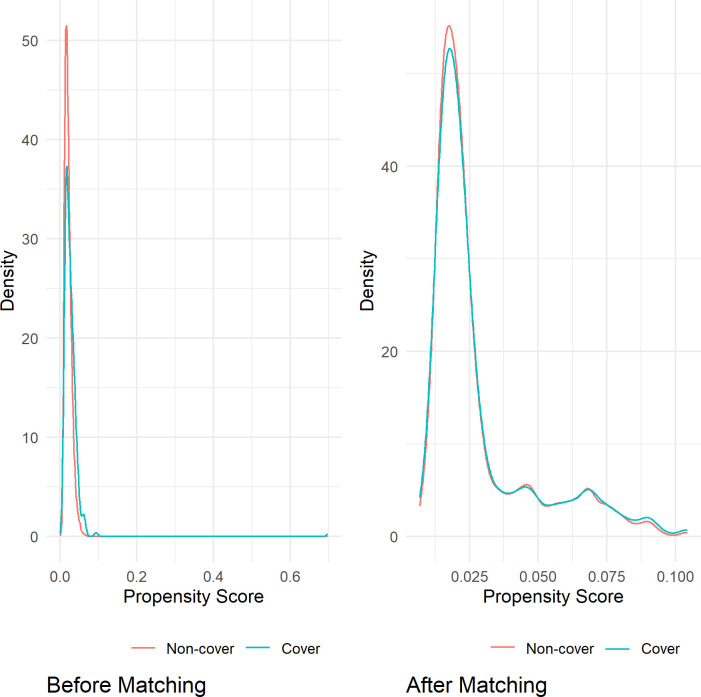
The kernel density of propensity score for the cover and non-cover groups before and after matching for the dataset analyzing AAS from 2011 to 2017.

**Fig 4:**
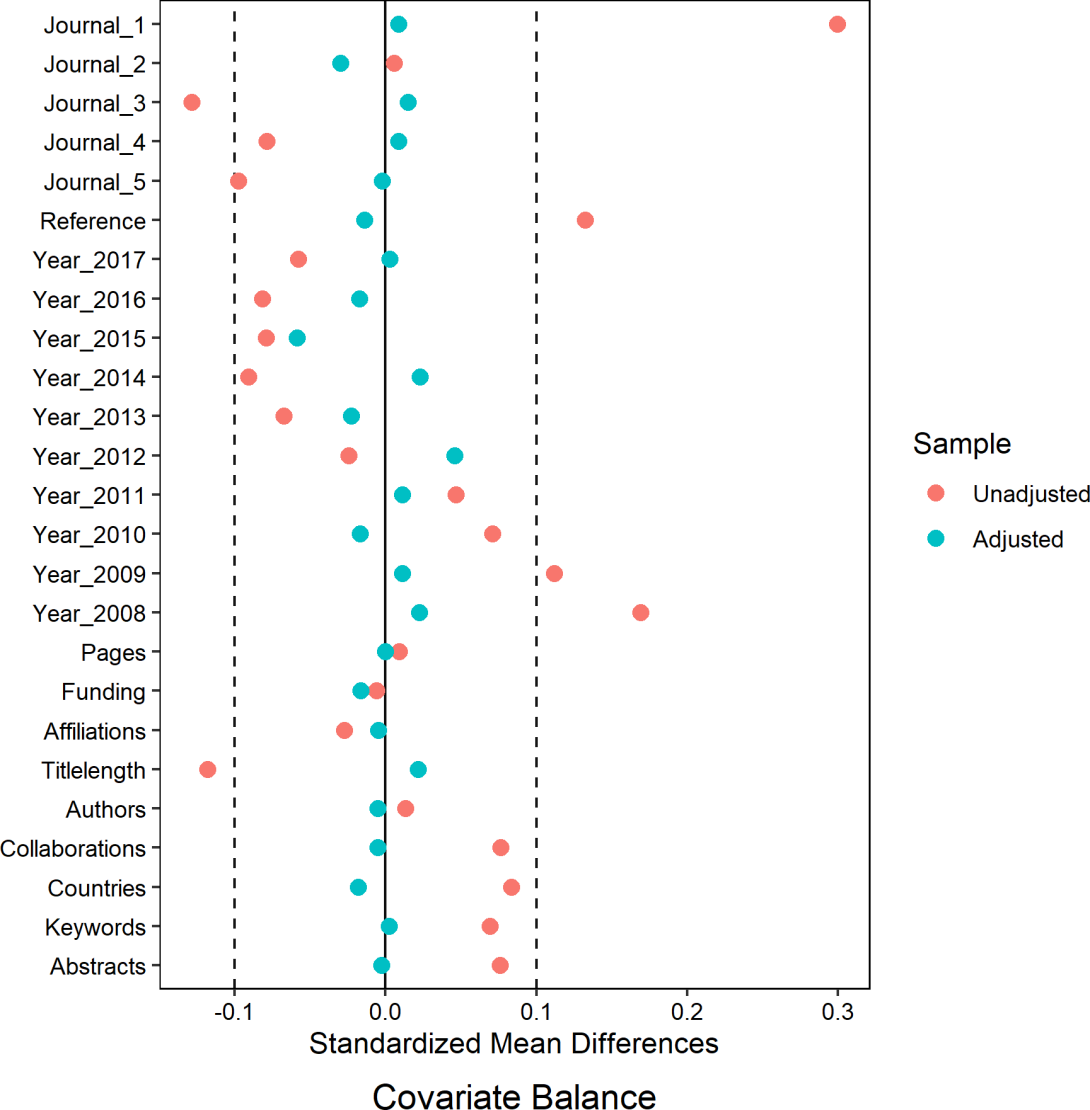
The visualization of standardized mean difference (SMD) before and after PSM for the dataset analyzing citations and readers from 2008 to 2017.

**Fig 5:**
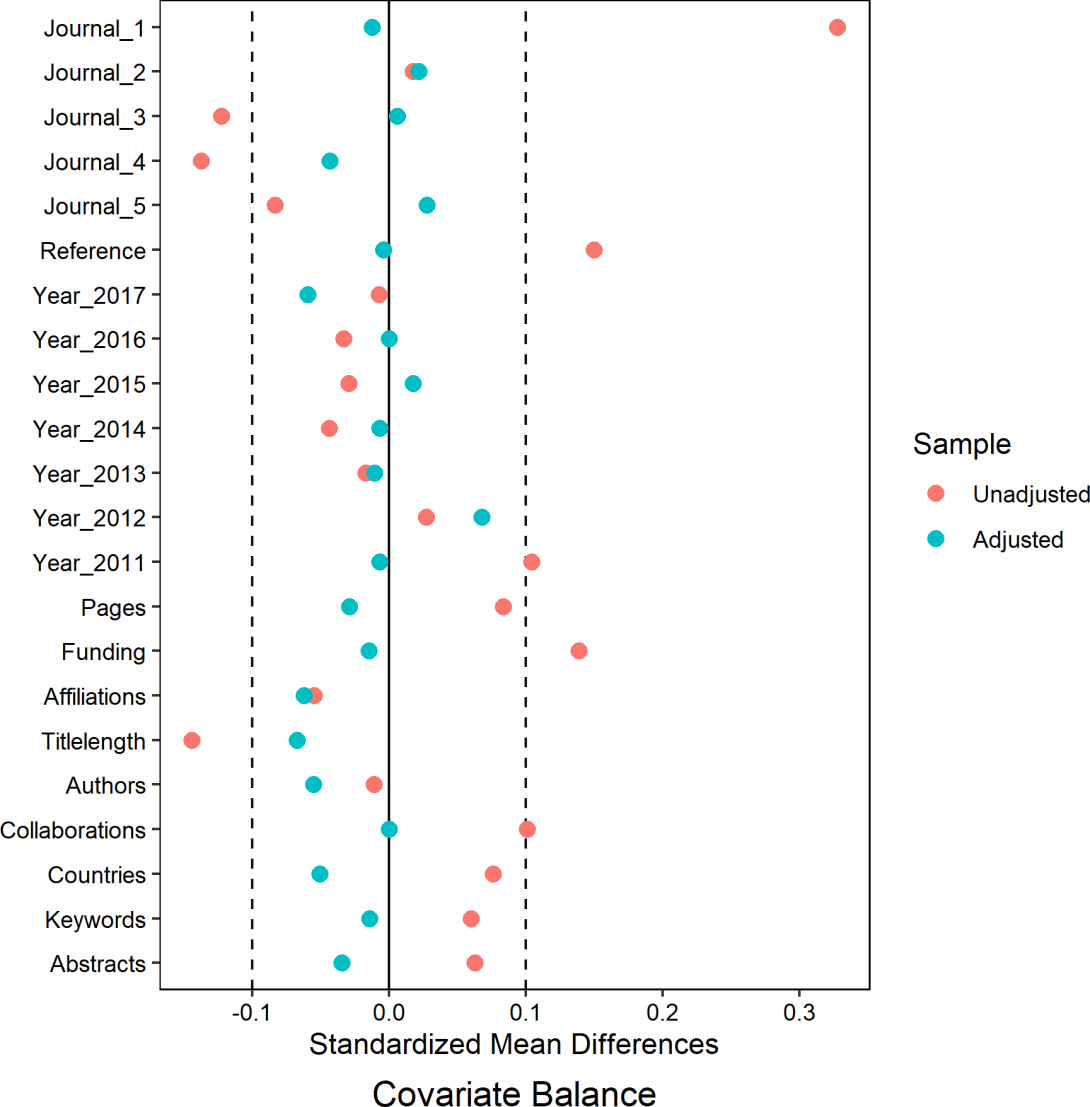
The visualization of standardized mean difference (SMD) before and after PSM for the dataset analyzing AAS from 2011 to 2017.

Cover and non-cover papers in each specialty were matched based on 12 variables (source journal, publication year, funding and collaborations, number of references, pages, keywords, abstracts, affiliations, authors, countries, and title length). For the dataset analyzing citations and readers from 2008 to 2017, 1,760 papers were matched, including 590 cover papers and 1,170 non-cover papers. In the dataset for analyzing AAS from 2010 to 2017, 1,212 papers were matched, consisting of 407 cover papers and 805 non-cover papers. Tables 3 and 4 showed the balance between the 12 variables after propensity matching. The overlaid density plots (Figs 2 and 3) illustrated the kernel density of propensity score before and after matching for both cover and non-cover papers. Meanwhile, Figs 4 and 5 depicted the standardized mean difference (SMD) before and after propensity score matching (PSM), providing visual evidence of the effectiveness of the PSM technique.

**Table 3:** Characteristics of cover and non-cover papers after PSM for the dataset analyzing citations and readers from 2008 to 2017.

Variable	Overall	Cover	Non-cover	Statistics	*P*
Total	1,760	590	1,170		
Year (%)				3.00	0.964
2008	169 (9.60)	60 (10.17)	109 (9.32)		
2009	169 (9.60)	59 (10.00)	110 (9.40)		
2010	181 (10.28)	59 (10.00)	122 (10.43)		
2011	172 (9.77)	59 (10.00)	113 (9.66)		
2012	155 (8.81)	57 (9.66)	98 (8.38)		
2013	188 (10.68)	60 (10.17)	128 (10.94)		
2014	163 (9.26)	57 (9.66)	106 (9.06)		
2015	204 (11.59)	61 (10.34)	143 (12.22)		
2016	180 (10.23)	58 (9.83)	122 (10.43)		
2017	179 (10.17)	60 (10.17)	119 (10.17)		
Journal (%)				0.55	0.969
PLOS Biology	332 (18.86)	115 (19.49)	217 (18.55)		
PLOS Computational Biology	367 (20.85)	118 (20.00)	249 (21.28)		
PLOS Genetics	353 (20.06)	120 (20.34)	233 (19.91)		
PLOS Neglected Tropical Diseases	347 (19.72)	117 (19.83)	230 (19.66)		
PLOS Pathogens	361 (20.51)	120 (20.34)	241 (20.60)		
Reference (median (IQR))	58 (31)	57 (30.75)	58 (30.75)	−0.71	0.476
Pages (median (IQR))	14 (8)	14 (8)	14 (8)	−0.29	0.77
Keywords (median (IQR))	10 (0)	10 (0)	10 (0)	−0.37	0.708
Abstracts (median (IQR))	242 (90.25)	242 (87)	242 (94)	−0.54	0.592
Funding (%)	1640 (93.18)	548 (92.9)	1092 (93.3)	0.07	0.799
Affiliations (median (IQR))	4 (5)	4 (5)	4 (5)	−0.70	0.481
Titlelength (median (IQR))	13 (6)	13 (6)	13 (6)	−0.51	0.614
Authors (median (IQR))	6 (5)	6 (5)	6 (5)	−0.42	0.673
Collaborations (%)	892 (50.68)	299 (50.7)	593 (50.7)	<0.001	0.998
Countries (median (IQR))	2 (1)	2 (1)	2 (1)	−0.04	0.967

Count variables were represented using case numbers and percentages, continuous variables were described using the median (interquartile range).

PSM = propensity score matching, IQR = interquartile range.

**Table 4:** Characteristics of cover and non-cover papers after PSM for the dataset analyzing AAS from 2011 to 2017.

Variable	Overall	Cover	Non-cover	Statistics	*P*
Total	1,212	407	805		
Year (%)				2.04	0.916
2011	168 (13.86)	57 (14.00)	111 (13.79)		
2012	152 (12.54)	57 (14.00)	95 (11.80)		
2013	180 (14.85)	59 (14.50)	121 (15.03)		
2014	168 (13.86)	56 (13.76)	112 (13.91)		
2015	174 (14.36)	60 (14.74)	114 (14.16)		
2016	173 (14.27)	58 (14.25)	115 (14.29)		
2017	197 (16.25)	60 (14.74)	137 (17.02)		
Journal (%)				0.68	0.954
*PLOS Biology*	223 (18.40)	76 (18.67)	147 (18.26)		
*PLOS Computational Biology*	239 (19.72)	82 (20.15)	157 (19.50)		
*PLOS Genetics*	250 (20.63)	84 (20.64)	166 (20.62)		
*PLOS Neglected Tropical Diseases*	257 (21.20)	81 (19.90)	176 (21.86)		
*PLOS Pathogens*	243 (20.05)	84 (20.64)	159 (19.75)		
Reference (median (IQR))	59 (30)	58 (32)	59 (29)	−0.21	0.834
Pages (median (IQR))	16 (9)	16 (9)	16 (9)	−0.19	0.848
Keywords (median (IQR))	10 (0)	10 (0)	10 (0)	−0.40	0.686
Abstracts (median (IQR))	245 (92)	242 (87.50)	246 (94)	−0.65	0.514
Funding (%)	1178 (97.19)	395 (97.05)	783 (97.27)	0.00	0.976
Affiliations (median (IQR))	4 (5)	4 (4.50)	4 (6)	−0.70	0.487
Titlelength (median (IQR))	13 (7)	13 (6)	13 (7)	−0.77	0.439
Authors (median (IQR))	6 (6)	6 (5)	7 (6)	−0.70	0.482
Collaborations (%)	636 (52.48)	214 (52.58)	422 (52.42)	0.003	0.959
Countries (median (IQR))	2 (1)	2 (1)	2 (1)	−0.21	0.837

Count variables were represented using case numbers and percentages, continuous variables were described using the median (interquartile range).

PSM = propensity score matching, IQR = interquartile range, AAS = Altmetric attention score.

### Nonparametric analysis after PSM

This study employed a nonparametric test to compare the impact and attention garnered by cover papers and non-cover papers following Propensity Score Matching (Table 5). Results showed that cover papers gained higher citations (*P* < 0.001), much more readers (*P* < 0.001), and higher AAS (*P* < 0.001) than non-cover papers.

**Table 5:** The result of the Wilcoxon signed-rank test after PSM.

Variable	Cover median (IQR)	Non-cover median (IQR)	Statistics	*P*
**Readers**	101.50 (116.75)	89 (99.75)	528	**<0.001**
**Citations**	59 (77)	51 (73.25)	560	**<0.001**
**AAS**	12 (34)	5 (14)	1384	**<0.001**

IQR = interquartile range, AAS = Altmetric attention score.

### Sensitivity analysis

To ensure the robustness of the results, this study employed various matching methods to test the sensitivity of the findings, including 1:1, 1:3, and 1:4 nearest neighbor caliper matching, radius matching, and kernel matching. Among these, radius and kernel matching failed the balance diagnostics and were consequently excluded from the analysis. The PSM results that met the balance diagnostics were presented in Table 6. Across the different matching methods, all three metrics for cover and non-cover papers passed significance tests, indicating strong robustness of the results.

**Table 6:** The result of sensitivity analysis.

Ratio	Variable	Cover median (IQR)	Non-cover median (IQR)	*P*
1:1	Citations	59 (77)	50 (69)	<0.001
	Readers	102 (116)	79 (96)	<0.001
	AAS	12 (34)	6 (16)	<0.001
1:3	Citations	59 (77)	49 (74)	<0.001
	Readers	102 (116)	84 (96)	<0.001
	AAS	12 (34)	6 (14)	<0.001
1:4	Citations	59 (77)	50 (72)	<0.001
	Readers	102 (116)	84 (93)	<0.001
	AAS	12 (34)	5 (13)	<0.001

IQR = interquartile range, AAS = Altmetric attention score.

### Regression analysis

This study conducted further linear regression analysis to verify the above results. The explanatory variable was the type of paper (cover or non-cover), the 12 variables mentioned in PSM were incorporated as control variables. Initially, this study performed a univariate linear regression, selecting variables that demonstrated significant differences for subsequent exploration in a multivariate linear regression analysis. Results were presented in Table 7.

**Table 7:** The results of univariate and multivariate linear regression for variables affecting citations, readers and AAS.

Variable	Citations				Readers				AAS			
	Univariate analysis		Multivariate analysis		Univariate analysis		Multivariate analysis		Univariate analysis		Multivariate analysis	
	*b*	*P*	*b*	*P*	*b*	*P*	*b*	*P*	*b*	*P*	*b*	*P*
Cover	**0.150**	**0.029**	**0.145**	**0.025**	**0.166**	**0.017**	**0.174**	**0.009**	**0.685**	**<0.001**	**0.691**	**<0.001**
Year												
2017	Ref		Ref		Ref		Ref		Ref		Ref	
2016	0.229	0.111	0.234	0.084	0.157	0.280	0.160	0.254	−0.093	0.512	−0.132	0.288
2015	0.325	0.020	0.305	0.020	0.263	0.062	0.250	0.066	−0.546	<0.001	−0.588	<0.001
2014	0.426	0.004	0.305	0.051	0.251	0.093	0.036	0.822	−0.593	<0.001	−0.883	<0.001
2013	0.395	0.006	0.351	0.020	0.174	0.226	0.016	0.920	−1.001	<0.001	−1.264	<0.001
2012	0.508	0.001	0.473	0.003	0.264	0.081	0.105	0.515	−1.266	<0.001	−1.552	<0.001
2011	0.563	0.000	0.561	<0.001	0.229	0.119	0.085	0.591	−1.398	<0.001	−1.698	<0.001
2010	0.326	0.023	0.288	0.060	−0.042	0.771	−0.221	0.160	–	–	–	–
2009	0.502	0.001	0.455	0.004	0.020	0.892	−0.149	0.354	–	–	–	–
2008	0.373	0.011	0.310	0.048	−0.129	0.384	−0.337	0.036	–	–	–	–
Journal												
*PLOS Biology*	Ref		Ref		Ref		Ref		Ref		Ref	
*PLOS Computational Biology*	−0.849	<0.001	−0.749	<0.001	−0.624	<0.001	−0.583	<0.001	−1.084	<0.001	−1.174	<0.001
*PLOS Genetics*	−0.239	0.017	−0.294	0.003	−0.432	<0.001	−0.482	<0.001	−1.098	<0.001	−1.068	<0.001
*PLOS Neglected Tropical Diseases*	−0.971	<0.001	−0.885	<0.001	−0.816	<0.001	−0.816	<0.001	−1.452	<0.001	−1.699	<0.001
*PLOS Pathogens*	−0.263	0.008	−0.235	0.017	−0.555	<0.001	−0.544	<0.001	−1.016	<0.001	−0.955	<0.001
Reference	0.008	<0.001	0.006	<0.001	0.007	<0.001	0.007	<0.001	0.004	0.023	0.005	0.001
Pages	0.010	0.035	−0.002	0.759	0.011	0.031	−0.018	0.023	0.040	<0.001	−0.025	0.003
Keywords	0.087	<0.001	0.012	0.506	0.009	0.573	–	–	−0.056	0.007	−0.072	0.000
Abstracts	0.001	0.072	–	–	0.000	0.524	–	–	−0.002	0.003	0.001	0.013
Funding	0.023	0.856	–	–	0.111	0.397	–	–	−0.121	0.630	–	–
Affiliations	0.026	<0.001	−0.007	0.394	0.022	<0.001	0.002	0.775	0.018	0.033	−0.008	0.308
Titlelength	−0.021	0.008	−0.027	0.001	−0.033	<0.001	−0.032	<0.001	−0.044	<0.001	−0.033	0.000
Authors	0.032	<0.001	0.026	0.001	0.021	<0.001	0.015	0.064	0.011	0.127	–	–
Collaborations	0.243	<0.001	0.202	0.011	0.251	<0.001	0.222	0.007	0.277	0.001	0.213	0.018
Countries	0.109	<0.001	0.023	0.489	0.095	<0.001	0.015	0.665	0.105	<0.001	0.098	0.005

*b*: the unstandardized coefficients.

The univariate analysis revealed that, without considering control variables, being featured on the cover positively associated with citations (*b* = 0.150, *P* = 0.029), readers (*b* = 0.166, *P* = 0.017), and AAS (*b* = 0.685, *P* < 0.001). After controlling for variables, multiple regression still demonstrated a positive correlation. The regression coefficients exhibited weak positive relationships between “being cover” and citations (*b* = 0.145, *P* = 0.025), AAS (*b* = 0.691, *P* < 0.001), and Mendeley readers (*b* = 0.174, *P* = 0.009) for sample papers in PLOS journals. Indicating that, holding other variables constant, if a paper is featured as a cover, Ln(1 + citations), Ln(1 + AAS), and Ln(1 + readers) will increase by an average of 0.145, 0.691, and 0.174, respectively.

## Discussion

In this study, we aimed to investigate the impact and visibility of cover papers in five PLOS journals, all of which are open-access publications from the same publisher, utilizing the PSM method. It is important to note that while these PLOS journals do not possess exceptionally high impact factors, they maintain a consistent level of influence within their respective fields. One intriguing finding is that being featured on the cover of a journal may statistically positively associated with an article’s citations, readers and AAS, indicating enhanced visibility and academic impact. While, this relationship appears to be weak, and this correlation does not necessarily imply causality Therefore, the findings may not be generalizable to broader contexts.

Previous research in this area has commonly utilized citation frequency. However, the methodology and results of this study differ from previous works. This study employed citations from Dimensions, whereas prior studies relied on citations from WOS [6–8] and Scopus [10]. Consistent with recent findings [6,8,9], this study revealed a significant disparity in citation counts between cover papers and non-cover papers published by the same publisher. Conversely, Wang et al. [7] found no difference in citations for cover and non-cover papers in PLOS Biology (one of the journals included in this study) from 2006 to 2010. The use of different datasets yielded varying results, further corroborating this study’s finding that the correlation between being featured and citation frequency is minimal. Additionally, propensity score matching was employed to mitigate certain confounding factors in this analysis.

Cover papers are typically featured on the front cover of journals, making them highly recognizable. This prominent placement attracts more reader attention and significantly increases the visibility and exposure of the paper. Meanwhile, the higher citations for cover papers suggest, to some extent, a superior average quality compared to non-cover papers. The factors associated with citation frequency can be categorized into novelty, bibliometric, and academic-network factors, with novelty factors exerting a greater influence on citation counts than other categories [48]. This underscores the complexity of academic impact. Furthermore, this study found that being featured as a cover paper was weakly correlated with the academic influence of a research paper. Therefore, further in-depth research is necessary to investigate whether factors such as novelty, technical reproducibility, and interdisciplinary relevance influence the citation frequency of cover papers.

Altmetrics have been extensively employed to assess the impact of research within the social media landscape [49]. There are two types of Altmetric.com scores, the attention score AAS and the readers score [50], which are classified as social attention and scholarly attention respectively in this study.

On one hand, this study suggested that cover papers, as indicated by their higher AAS, garnered significantly more social attention than non-cover papers. This finding corroborated the results of Kong and Wang [6], the only previous study comparing AAS between cover and non-cover papers. However, the two studies differed in several methodological aspects: (1) This study analyzed papers from five PLOS journals published between 2008 and 2017, while Kong and Wang [6] focused on papers from the prestigious journal *Nature* published from 2011 to 2015; (2) The regression coefficient for the ‘cover’ variable and AAS in this study was estimated to be positive and significant at the 0.001 level; (3) This study employed PSM to control for confounding factors such as discipline, funding, international collaboration, and the number of countries involved. Among various altmetric indicators, including Facebook, Google + , CiteULike, Mendeley, Wikipedia, and other online blogs, Twitter emerged as the preferred platform for scholars and researchers to share and disseminate their work and opinions on research. The elevated AAS scores were primarily attributed to a greater number of tweets. This finding suggests that heightened visibility may be linked to the prominent display of these papers on journal covers, which effectively captures public interest and increases media exposure. The correlation between cover status and AAS highlights the relationship between journal covers and research dissemination and societal impact.

On the other hand, Mendeley reader counts were considered to scrutinize academic attention in this study. Mendeley can help manage references, store and organize research data, collaborate online with others, and find relevant research [51]. However, one study reported that Mendeley users tend to prefer reading articles authored within their own country, often overlooking works from certain other countries [52]. The quantity of Mendeley readers, which is one of the metrics provided by Altmetric.com, has been regarded as an alternative measure of scholarly output for evaluating research by scientists [53–56]. Notably, no prior study has examined Mendeley readers in comparing the effectiveness of cover versus non-cover papers. It is important to note that adding a paper to Mendeley does not necessarily equate to its citation, nor does it guarantee that a referencing article will ultimately be published [12]. Nonetheless, this does indicate that the researcher has shown interest in the article. Furthermore, reader engagement can vary significantly across different disciplines [57].

This study showed a positive correlation between being featured as a cover paper and readership; however, it’s important to note that this relationship was relatively weak. It is evident that cover papers possess their own merits, and while they may attract some initial attention, scholars’ primary focus remains on the substantive content of the articles. Unlike social attention, which can be influenced by visual prominence and media coverage, academic readership is driven more by the substantive content and utility of the research to scholarly communities. Researchers and academics typically prioritize papers that contribute directly to their fields of study, irrespective of whether they are featured on a journal’s cover. Even if a paper is featured on the cover, the quality and relevance of the content will ultimately determine whether readers engage with it by saving or citing it.

### Limitations and suggestions for future research

This study also had several limitations. (1) Only papers published in 5 OA PLOS journals representing different disciplines were collected. Thus, the findings might not be generalizable to other specialized or diverse research disciplines. (2) Social attention only considered AAS, detailed mentions in social media (e.g., Twitter, Facebook, News, Blogs) in Altmetric.com were not considered. Thus, we could not find which platforms the activities related to cover or non-cover papers occurred. (3) For the Mendeley readership, only the number of readers was considered, ignoring the geographic, categories and professions of readers. (4) Although the study used PSM to control for certain factors, it’s essential to recognize that the impact of academic papers is influenced by a multitude of variables. This study included only some quantifiable indicators, omitting crucial qualitative factors, such as author and institutional reputation, as well as the novelty, originality, and micro themes of the paper. This omission notably restricts the generalizability of the findings. (5) While PSM effectively balances observed covariates and reduces bias stemming from these observed factors, it is crucial to acknowledge that PSM, inherent to all observational studies, cannot eliminate the potential for unobserved confounding. Consequently, the findings are ultimately indicative of correlation rather than definitive causality. (6) The estimated average treatment effect of the treated (ATT) from PSM applies specifically to the treated group within the study sample and may not generalize to broader papers or different research contexts. And the choice of variables in the propensity score model can influence the estimated ATT. Future research could expand the scope by including multidisciplinary journals, non-OA journals, and journals with varying impact factors. And a broader range of variables should be incorporated to achieve a more comprehensive understanding of the impact of the cover paper. If possible, author-level variables (such as the h-index, author reputation, collaboration network, university ranking, institution’s reputation, and funding levels), and paper-level variables (such as surveys of researchers in the field to rate the novelty and originality of the paper, natural language processing techniques for analyzing the paper’s content, and collaboration across disciplines) could be included in the future research.

## Conclusions

Based on the metric datasets of papers from 5 PLOS journals and propensity score matching strategy, this study revealed a statistically weak positive correlation between being designated as a cover paper with the academic impact and attention received by the paper, particularly in terms of societal attention (AAS). This indicated that cover papers may receive slightly more citations and attention compared to non-cover papers to some extent.

## Supporting information

S1 Data Data employed in this study before propensity score matching. (XLS)
